# Early Initiation of rhGH Therapy Significantly Improves Height Gain and Reduces the Gap to Target Height in Children Born Small for Gestational Age: A Multicenter Retrospective Study

**DOI:** 10.3390/children13050641

**Published:** 2026-05-03

**Authors:** Letteria Anna Morabito, Malgorzata Wasniewska, Cecilia Lugarà, Emanuela Pignatone, Domenico Corica, Renato Vaiasuso, Alessandra Cipriani, Giovanni Luppino, Roberto Coco, Giorgia Pepe, Tiziana Abbate, Stefano Stagi, Tommaso Aversa

**Affiliations:** 1 Department of Human Pathology in Adulthood and Childhood “Gaetano Barresi”, University of Messina, 98124 Messina, Italy; letteria.morabito@gmail.com (L.A.M.); malgorzata.wasniewska@unime.it (M.W.); domenico.corica@unime.it (D.C.); alessandra.cipriani@gmail.com (A.C.); cocoroberto93@gmail.com (R.C.); giorgiapepe23@gmail.com (G.P.); tiziana.abbate93@gmail.com (T.A.); tommaso.aversa@unime.it (T.A.); 2 Department of Health Sciences, University of Florence, 50139 Florence, Italy; emanuela.pignatone@unifi.it (E.P.); renato.vaiasuso@unifi.it (R.V.); stefano.stagi@unifi.it (S.S.); 3 Auxoendocrinology Center, Anna Meyer Children’s Hospital, 50139 Florence, Italy

**Keywords:** small for gestational age, growth trajectory, height gain, adult height, recombinant human growth hormone

## Abstract

**Highlights:**

**What are the main findings?**

- Recombinant human growth hormone (rhGH) significantly improves growth trajectory in children born small for gestational age (SGA).
- The greatest height gain occurs during the prepubertal phase, while pubertal growth contributes less to adult height attainment.

**What is the implication of the main finding?**

- Early initiation of rhGH treatment is essential to optimize adult height outcomes in SGA patients.
- Clinical management should prioritize maximizing prepubertal growth and appropriate timing to refer short SGA children to endocrinological evaluation.

**Abstract:**

**Background**: Treatment with recombinant human growth hormone (rhGH) is approved for children born small for gestational age (SGA) who fail to show postnatal catch-up growth; however, optimizing its efficacy remains a challenge. **Aim**: to evaluate the impact of rhGH therapy on growth trajectory (GT) and adult height (AH) in SGA children and to identify factors influencing height gain (HG). **Methods**: A total of 49 SGA children (24 males, 25 females) without postnatal growth recovery and treated with rhGH were enrolled. Clinical and anthropometric data were collected at treatment initiation (T0), after 1 (T1) and 2 years (T2) of therapy, at pubertal onset (P0), during the first (P1) and second year (P2) of puberty, and at attainment of AH. Parameters included age, bone age, H, weight, BMI (all expressed as SDS), HG, and the difference between H and target height (Δ H-TH). **Results**: a significant increase in HG at all evaluated stages was observed (*p* < 0.05). The H–TH difference progressively decreased from T0, particularly until the first two years of puberty. Nevertheless, mean AH was −1.75 ± 0.63 SDS, and it was found to fall within the TH range in 86% of cases. Univariate and multivariate regression analysis revealed that age and H at T0 were independent predictors of HG. **Conclusions**: rhGH treatment has a positive impact on GT in children born SGA. Pubertal growth has a limited contribution in influencing AH of these patients. H and timing of treatment initiation significantly influence HG in SGA children. Early selection of patients for rhGH therapy could further improve their GT.

## 1. Introduction

Persistent short stature is one of the most common complications in children born small for gestational age (SGA), as nearly 15% of them have compromised adult height (AH). Literature data show that approximately 10–15% of SGA infants do not show satisfactory growth recovery and may have a negative impact on AH [1].

The average AH of SGA infants with adequate catch-up growth has been estimated at around −0.7 SDS, while the average AH of SGA infants without growth recovery is lower, at around −1.7 SDS [2].

It has been observed that SGA infants with a height (H) below −2 SDS at 3 years of age have a 7-fold increased risk of remaining short as an adult, while a birth weight below −2 SDS is associated with a 5-fold increased risk of short stature in adulthood [2,3,4].

The reasons for reduced/absent catch-up growth in children born SGA are not well understood. Factors that positively influence growth in early age include taller parents, female sex, and faster length gain during the first years of life [5,6].

Treatment with recombinant human growth hormone (rhGH) has been approved for SGA infants with short stature, due to inadequate or absent postnatal catch-up growth during the first years of life, to improve AH [7,8,9].

The main goal of rhGH therapy is to increase H and achieve an AH in line with growth potential [9], but positive influences on metabolic profile, blood pressure, and body composition have also been described [8,10,11].

Therefore, the positive impact of rhGH treatment is not limited to growth parameters and improvements in AH, but also extends to future health-related quality of life (HRQoL): it has been established that small SGA patients who do not receive rhGH treatment may experience a long-term decline in HRQoL [12].

Data on treatment efficacy show that height gain (HG) during rhGH therapy depends mainly on early initiation of therapy, an appropriate dosage regimen and high mid parental H [13].

Treatment with rhGH can be started from the age of 4 in Europe, while it is permitted from the age of 2 for formulations authorized in the United States and Latin America [14,15].

Despite the now well-established benefits, the response to rhGH therapy remains highly variable among SGA patients. Although the influence of pubertal growth on the outcome of AH in SGA children treated with rhGH has been the subject of debate over time, it has not yet been fully clarified [13,16].

Children born SGA may exhibit distinctive features of pubertal development. Although the onset of puberty (P0) generally occurs within the normal physiological age range, a tendency towards a relatively earlier age at puberty and a rapid progression compared with peers born with a weight appropriate for gestational age (AGA) has been described. This earlier onset of puberty is likely related to the metabolic imbalances observed in postnatal growth, with accumulation of visceral fat and consequent insulin resistance and hyperinsulinemia, which in turn are believed to play a key role in the development of a hyperandrogenic state, particularly in SGA girls [17,18].

Furthermore, pubertal growth in SGA individuals may be characterized by a reduced magnitude and shorter duration of peak H velocity, potentially limiting the contribution of puberty to total height gain (THG) and influencing AH outcomes [2,5].

The aim of our study is to evaluate growth trajectory (GT) and AH in children born SGA and treated with rhGH. Our secondary aim is also to identify factors influencing HG, including the impact of pubertal growth, and the H outcome of short SGA patients.

## 2. Materials and Methods

This study was designed as a multicenter, retrospective review of medical records of 49 children (25 females, 24 males) born SGA with short stature due to unsatisfactory catch-up growth, followed at 2 Italian Pediatric Endocrinology Centers (Pediatric Endocrinology Centre of the Gaetano Martino University Hospital in Messina and Pediatric Endocrinology Unit of the Anna Meyer University Pediatric Hospital in Florence, Italy) from diagnosis until reaching AH and treated with rhGH, according to the current guidelines. The data were recorded and entered in a dedicated Microsoft Excel datasheet.

Inclusion criteria were as follows: (a) birth SGA regarding birth length or weight or both; (b) short stature (<−2.5 SDS), based on national growth references [19] at start of rhGH treatment; (c) normal perinatal course; (d) normal GH secretion; and (e) completion of rhGH therapy until the reaching of AH.

Only patients who had been followed in the same medical center from the start of rhGH therapy through to attainment of AH and consequently treatment discontinuation were included.

Exclusion criteria were (a) presence of chronic diseases that could impair growth during the follow-up time; (b) failing to attend routine follow-up visits; (c) presence of growth hormone deficiency (GHD) and/or multiple pituitary hormonal deficiency requiring hormonal replacement treatment; and (d) chromosomal anomalies, malformations or chondrodysplasias, and any other syndromes.

The diagnosis of SGA at birth was confirmed by retrieving neonatal data (birth weight and length) from neonatal medical records and comparing it with Italian neonatal reference tables [20].

SGA children were considered as of short stature if their H was below −2.5 SDS according to Italian growth standards specific for age and sex [19].

At baseline, data about family history of short stature and pubertal disorders (precocious/late puberty), perinatal history and auxological data at birth were collected.

Clinical, auxological, and biochemical data at the start and during the first 2 years of rhGH treatment (T0, T1, T2), at P0, at the first and second year of puberty (P1, P2), and at the reaching of AH were collected by tertiary pediatric endocrinologists during each semi-annual control visit to evaluate the GT. At each of these stages, the difference in SDS between the child’s H at the respective measurement and the family TH (delta H-TH: Δ H-TH) were also calculated.

Auxological data (H, weight, BMI) were expressed in cm and SDS for age and gender, based on Italian growth standards [19].

H of the patients and of their parents were measured with the Harpenden stadiometer. Bone age (BA) was determined according to Greulich et al. [21] and puberty was assessed according to Marshall and Tanner [22,23].

P0 was defined by the appearance of pubertal signs: breast buds in girls and testicular volume, assessed through Prader’s orchidometer, reaching 4 mL in boys. Precocious puberty was diagnosed when pubertal signs appeared before the age of 8 years in girls and 9 years in boys.

Weight was recorded using a mechanical column scale with sliding counterweights. BMI values were calculated using the formula: weight (kg)/H (m^2^).

Patients’ H was considered as adult when the increment was <1 cm over 1 year or when BA was 15 years in girls and 17 years in boys. AH adjusted to genetic target H (Δ AH-TH) SDS was calculated as the difference between AH SDS and the mean parental H (TH SDS).

TH was calculated using the formula: [father’s H + mother’s H]/2 ± 6.5 cm for male and female respectively, as described by Tanner et al. [24].

Patients’ AH was classified “within the TH” if the difference between their AH SDS and their TH SDS (Δ AH-TH) was no greater than ±1.5 SDS. Values below −1.5 SDS were classified as “below the TH range” and values above +1.5 SDS were classified as “above the TH range” [25,26].

HG was determined by subtracting the pretreatment H SDS from the H SDS recorded at each evaluation during treatment. Total HG (THG) from the start of rhGH treatment was calculated as the AH SDS minus H SDS at T0’. For the hormonal assays, blood samples were collected at 08:00 a.m. after overnight fasting.

Eligibility for rhGH therapy was established in accordance with Italian regulations (AIFA note 39) [27]. All the patients started therapy with rhGH after the fourth year of age if in the presence of a H below −2.5 SDS for age and sex, and a growth velocity below 0 SDS for age and sex.

During the follow-up rhGH dose, the dose was subsequently adjusted based on growth response and IGF-1 levels, in accordance with current clinical practice. rhGH treatment was maintained until the attainment of AH and/or as the attainment of adult BA, as previously defined [28,29]. Comparative analyses were also performed by stratifying the cohort into subgroups based on age at rhGH treatment initiation (before and after 7 years old split into subgroup A and subgroup B, respectively). No formal correction for multiple comparisons was applied due to the exploratory nature of the study and the relatively small sample size.

Finally, as no universally accepted definition of late treatment initiation exists, a post hoc analysis was performed and an exploratory cut off at 7 years was selected, corresponding to the point showing the greatest discrimination in H outcomes within our cohort. Additionally, the choice of this exploratory cutoff point also made it possible to adequately assess the extent of prepubertal growth during rhGH therapy.

### Statistical Analysis

Statistical analyses were performed using STATA/SE 19.0 for MacOS. A *p*-value lower than 0.05 was considered significant. The distribution of the variables was studied by the Kolmogorov–Smirnov test.

Categorical variables were expressed as absolute frequencies and percentage. Numerical variables were expressed as mean, SD, median and interquartile range (Q1–Q3).

The Wilcoxon signed rank test was adopted to compare the H (SDS) at baseline (T0) with values at T1, T2, P0, P1, P2 and AH (SDS) and to compare the Δ H-TH at baseline (T0) with values at T1, T2, P0, P1, P2 and at the reach of AH.

Parametric or non-parametric tests were adopted according to the distribution of the variables. Specifically, Student’s t test or the Mann–Whitney U test was applied with numerical parameters to identify possible differences between those stratified by sex and those by age at start of rhGH therapy.

The Spearman correlation test was used to assess the relationship between HG and variables of interest.

Finally, univariate and multivariate regression analyses were performed to identify potential factors associated with HG.

Post hoc power was estimated based on the observed difference in THG between the groups, using the pooled standard deviation and a two-sided significance level of α = 0.05.

## 3. Results

### 3.1. Overall Characteristic of the Study Population

We enrolled 49 patients (51% females and 49% males). The characteristics of the population at the baseline are shown in Table 1. All patients (100%) underwent rhGH treatment.

Family history of short stature was reported in 43% of the cohort, while 10% had a family history of precocious puberty, and 26.5% had a family history of delayed puberty.

In total, 81.6% of patients were born from physiologically normal pregnancies, while 24.5% of these were complicated by conditions such as pre-eclampsia, oligohydramnios, gestational diabetes and placenta previa. Vaginal delivery occurred in 39% of cases, cesarean section in 51% of cases and 10% had no recorded birth outcome. The mean gestational age was 37.6 ± 3.0 weeks, with a mean birth weight of 2.1 ± 0.6 kg (−1.96 ± 0.93 SDS) and a mean birth length of 44.2 ± 2.8 cm (−2.34 ± 0.83 SDS). A total of 28% of the patients were classified as SGA for birth length, 36% for birth weight and 36% for both.

The mean maternal H was 155.3 ± 6 cm (−1.08 ± 1.68 SDS), and the mean paternal H was 168.6 ± 7 cm (−0.97 ± 0.83 SDS) resulting in an average TH of −1.23 ± 0.73 SDS. Maternal menarche occurred at a mean age of 12.3 ± 1.5 years.

Short stature was diagnosed in SGA children at a mean age of 5.6 ± 2.9 years. rhGH therapy was started at a mean age of 7.3 ± 2.9 years and a mean dose of 0.035 ± 0.01 (µg /kg/day). The starting dose was determined in accordance with AIFA Note No. 39, which does not permit the use of higher doses in SGA infants [27].

The mean BA at T0’ was 6.3 ± 2.9 years.

At the rhGH therapy start, the mean H was −3.08 ± 0.56 SDS, the mean weight −3.25 ± 2.21 SDS and BMI was −1.20 ± 1.05 SDS. The duration of puberty was 3.14 ± 1.1 years.

### 3.2. Growth and Biochemical Outcomes During Follow-Up

Auxological parameters, including H, weight, BMI and Δ H-TH during the follow-up are summarized in Table 2.

Laboratory results during follow-up, including IGF-1 levels and glycemic metabolism (fasting glucose, insulin and HOMA-IR) are summarized in Table 3.

### 3.3. GT and AH

**Figure 1:**
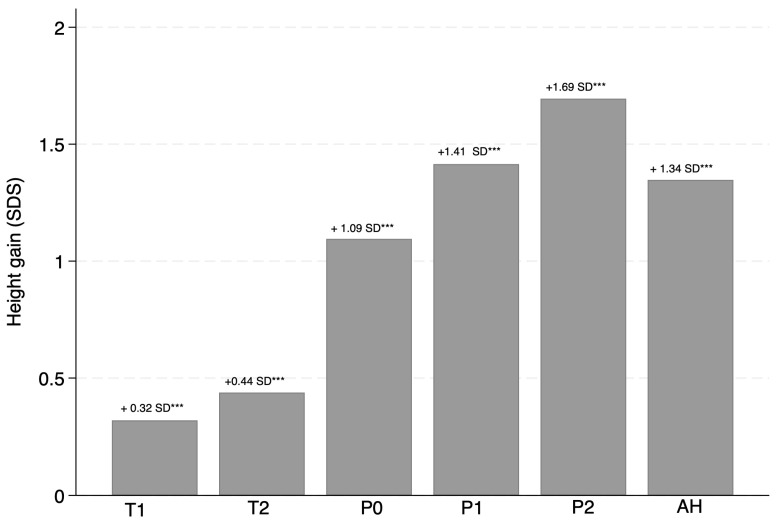
Mean (±SD) height gain from the baseline height (T0) at each follow-up step. Abbreviations: T0: start of rhGH treatment; T1: one year after the start of rhGH therapy; T2: two years after the start of rhGH therapy; P0: onset of puberty; P1: one year after the onset of puberty; P2: two years after the onset of puberty; AH: adult height. *** = *p* < 0.001.

**Figure 2:**
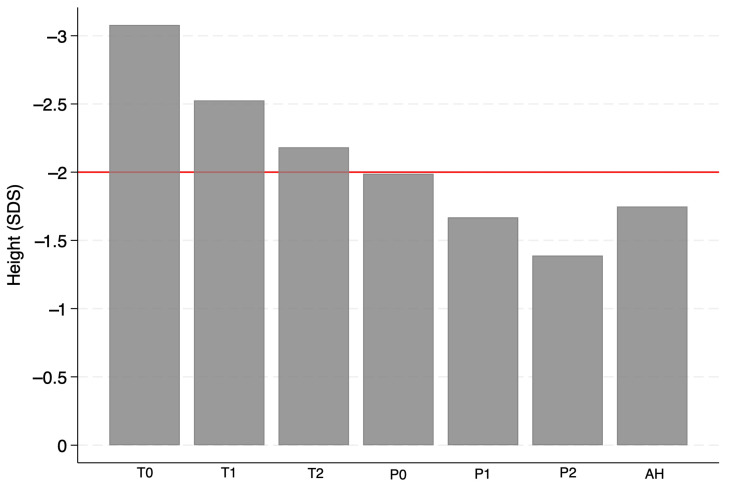
Mean (±SDS) height from baseline (T0) to AH. Abbreviations: T0: start of rhGH treatment; T1: one year after the start of rhGH therapy; T2: two years after the start of rhGH therapy; P0: onset of puberty; P1: one year after the onset of puberty; P2: two years after the onset of puberty; AH: adult height.

HG of patients treated with rhGH was consistent from baseline (T0’) at each follow-up stage, with a gain that remained significant at every stage of this investigation (*p* < 0.05), as shown in Figure 1 and Figure 2. Specifically, the mean HG from T0 to AH was +1.35 ± 0.67 SDS (*p* < 0.001).

The greatest increase in H occurred between the start of rhGH therapy (T0’) and the onset of puberty (P0), with a gain of 1.09 ± 0.75 SDS (*p* < 0.001). Although HG continued from P0 to AH, it was smaller, amounting to 0.25 ± 0.54 SD (*p* = 0.0027).

**Figure 3:**
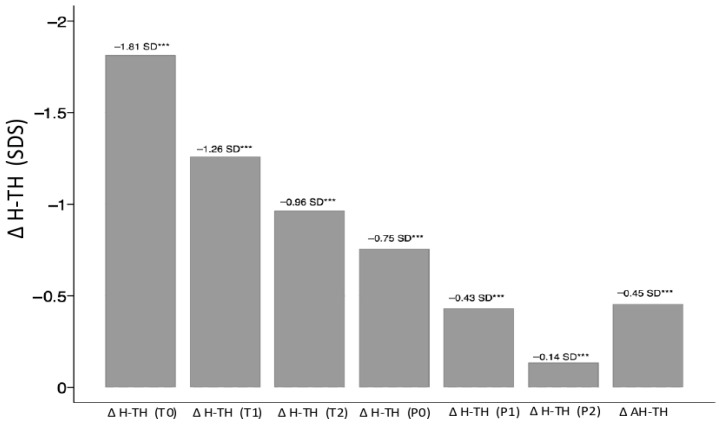
Mean (±SDS) Δ H-TH from baseline (T0) to AH. Abbreviations: Δ H-TH: difference in SD between the child’s height and the family target height; T0: start of rhGH therapy; T1: one year after the start of rhGH therapy; T2: two years after the start of rhGH therapy; P0: onset of puberty; P1: one year after the onset of puberty; P2: two years after the onset of puberty; AH: adult height. *** = *p* < 0.001.

Furthermore, when the H of patients was evaluated in comparison with the TH, the difference (Δ H-TH) decreased progressively at each stage (*p* < 0.05), as illustrated in Figure 3.

Specifically, Δ H-TH decreased progressively from baseline (T0) to T2, from −1.81 ± 0.80 to −0.96 ± 0.82 SDS, representing a gain of 0.85 ± 0.53 SD (*p* < 0.001); to P0, to −0.75 ± 0.90 SDS, representing a gain of 1.07 ± 0.87 SDS (*p* < 0.001); and finally to P2, to −0.14 ± 1.18 SDS, representing a gain of 1.69 ± 1.09 SDS (*p* < 0.001). Overall, upon reaching AH, Δ H-TH was −0.45 ± 0.79 SDS, representing an overall recovery relative from T0 to AH (Δ H-TH) of +1.37 ± 0.68 SDS (*p* < 0.001).

At the end of the observation period, AH SDS was found to fall within the TH range in 86% of cases and remained below the target range in 14% of cases.

It was assessed whether earlier initiation of rhGH therapy was associated with greater benefits in terms of HG.

Overall, 53% of SGA children started rhGH therapy before the age of seven, at an average age of 5.09 ± 0.99 years (group A), while 47% initiated treatment at the age of seven or later, at an average age of 9.95 ± 1.98 years (group B).

**Figure 4:**
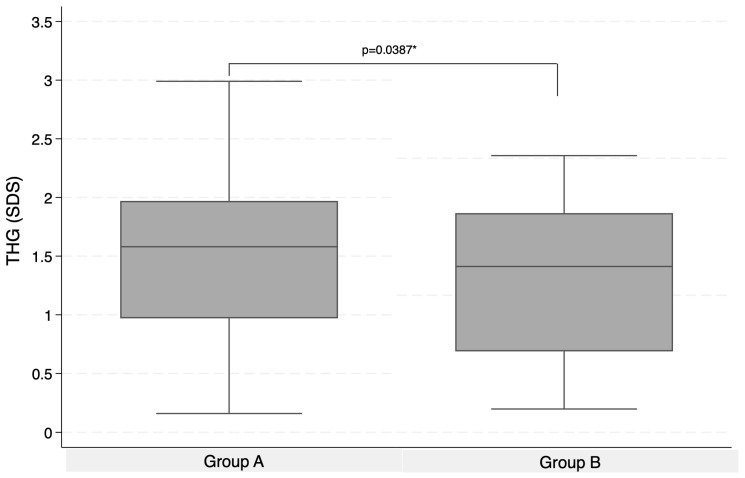
Total HG (mean ± SD) in group A and in group B. Abbreviations: THG: total height gain. * *p* < 0.05.

SGA children who started therapy before age of 7 achieved a significantly greater total HG than those who began therapy at 7 years old or later (+1.53 ± 0.14 SDS vs. +1.13 ± 0.57 SDS; *p* = 0.0387) (Figure 4).

In group A, girls constituted the majority (69%; *p* = 0.0073) and age at diagnosis of SGA-related short stature was earlier (3.87 ± 0.32 years) than in group B (7.65 ± 0.61 years) (*p* < 0.001).

Furthermore, children who started rhGH therapy before the age of 7 showed an earlier onset of puberty than those who started rhGH therapy at ≥7 years of age (9.57 ± 1.31 years versus 11.89 ± 0.32 years; *p* = 0.00).

H SDS was higher in group A compared with group B at P0 (−1.63 ± 0.48 SDS vs. −2.37 ± 0.58 SDS, *p* < 0.001), at P1 (−1.25 ± 0.59 SDS vs. −2.13 ± 0.67 SDS, *p* < 0.001) and at P2 (−0.95 ± 0.90 SDS vs. −1.86 ± 0.73 SDS, *p* < 0.001). Finally, there was also a trend towards significance for AH SDS, which was higher in SGA children who started therapy before the age of 7 (−1.59 ± 0.60 SDS vs. −1.93 ± 0.64, *p* = 0.059).

No differences were found between the two groups in H SDS at T0, T1 and T2, or in Δ H-THz and BMI SDS at the different follow-up stages.

### 3.4. Comparison of Outcomes for Sex

#### Comparison of Results by Sex

When the cohort was stratified by sex, no differences were found in H in SDS and BMI in SDS at T0, T1, T2, P0, P1, P2, FH, or total HG in SDS. In both females and males, H in SDS and Δ H-TH improved significantly compared to T0 at each follow-up stage (*p* < 0.001 for both sexes at all follow-up points). Specifically, in females, the improvement in H SDS between P0 and AH was not significant (*p* = 0.432), nor was the change in Δ H-TH between P0 and Δ H-TH at AH (*p* = 0.428).

Although no differences were found between the sexes in terms of age at diagnosis of short stature, females in our cohort started rhGH therapy at a younger age than males (4.99 ± 0.48 years vs. 8.72 ± 0.58 years; *p* < 0.001) and had an earlier onset of puberty than males (9.57 ± 0.26 years vs. 11.89 ± 0.32 years; *p* < 0.001). Despite the differences, H SDS at P0 was comparable between the two sexes.

No sex-related differences were found in IGF1 values or rhGH dose at the start of treatment and during follow-up.

### 3.5. Spearman Correlation Analysis

Spearman correlation analyses were performed to explore the associations between HG, AH and H SDS at P0 and selected variables (Table 4).

HG showed a significant inverse correlation with BA at T0 indicating that a more advanced baseline BA was associated with a lower HG achieved (r = −0.3548, *p* = 0.0342).

AH was positively correlated with TH (r = 0.4330, *p* = 0.0099), maternal H (r = 0.4964, *p* = 0.0027) and with H SDS at P0 (r = 0.4933, *p* = 0.0019). H SDS at P0 demonstrated a strong negative correlation with age at treatment initiation (T0) (r = −0.7320, *p* < 0.001), BA at T0 (r = −0.7004, *p* < 0.001) and age at P0 (r = −0.4642, *p* = 0.0036). It was positively correlated with duration of rhGH treatment (r = 0.5788, *p* < 0.001).

No significant correlations were observed between HG, AH and H SDS at P0 and sex, gestational age, birth weight SDS, birth length SDS, maternal age at menarche, rhGH dose at T0, BMI at T0, and HG during the first and the second year of therapy.

### 3.6. Factors Influencing HG

- Univariate and multivariate regression analysis

Univariate regression analysis showed that HG was significantly associated with mother H SDS (β = 0.123, 95% CI 0.004 to 0.242, *p* = 0.042), age at rhGH initiation (β = −0.080, 95% CI −0.14 to −0.016, *p* = 0.014), BA at T0 (β = −0.081, 95% CI −0.145 to −0.016, *p* = 0.016), H SDS at T0 (β = −0.584, 95% CI −0.898 to −0.269, *p* = 0.001), IGF-1 levels at T0 (β = −0.003, 95% CI −0.01 to −0.000, *p* = 0.037), H SDS at P0 (β = 0.421, 95% CI: 0.14 to −0.70; *p* = 0.004), HG at P0 (β = 0.640, 95% CI: 0.451 to 0.828; *p* < 0.000) and duration of rhGH therapy (β = 0.132, 95% CI: 0.048 to 0.216; *p* = 0.003).

No significant associations were found with gestational age, sex, birth length, birth weight, TH, father H SDS, age at menarche in girls, rhGH dose at T0, age at P0, rhGH dose at P0, IGF1 levels at P0 and duration of puberty.

Multivariate regression analysis confirmed the significant influence of the age at rhGH initiation (β = −0.100, 95% CI −0.186 to −0.013, *p* = 0.026) and H SDS at T0 (β = −0.853, 95% CI −1.625 to −0.805, *p* = 0.032), independently of sex, TH, gestational age, birth length, birth weight, BMI at T0, and duration of puberty (see Table 5).

## 4. Discussion

rhGH treatment has been used for several years in short children born SGA to improve GT and AH.

In our retrospective study, we observed that short SGA children treated with rhGH showed a significant HG from treatment initiation through puberty and until achievement of AH. A significant increase in H SDS was observed at each follow-up step. The overall THG of +1.35 ± 0.67 SDS is consistent with previous reports describing an average gain of approximately 1.2–1.3 SDS in treated SGA populations [12].

To minimize confounding factors affecting growth, we selected a relatively homogeneous cohort, excluding patients with concomitant GHD and genetic conditions known to negatively impact growth in SGA children (e.g., chromosomal abnormalities and Silver–Russell syndrome) [12,30]. This approach strengthens the interpretation of our findings, allowing a more accurate evaluation of the effects of rhGH therapy in SGA children with growth failure due to absent/inadequate catch-up growth.

The timing of treatment initiation is recognized as a major determinant of rhGH effectiveness. International consensus guidelines recommend starting rhGH therapy early, ideally between 2 and 4 years of age in children with severe growth impairment (H < −2.5 SDS), as younger age at treatment initiation is associated with greater benefits [12]. In addition, TH, baseline H SDS, and the difference between chronological and BA at treatment start have been positively associated with AH outcome [31,32].

In our cohort, rhGH therapy was initiated at a relatively late age. This likely reflects the retrospective nature of the study, the strict Italian regulatory criteria for treatment eligibility (Nota 39), and the need for longitudinal observation to document reduced growth velocity before therapy approval [27]. Furthermore, the need to exclude alternative causes of short stature, including GHD and other endocrine disorders, may have contributed to additional delays.

This delay may have negatively influenced AH outcomes. Indeed, although a significant reduction in the Δ H-TH was observed throughout treatment, only a proportion of patients achieved an AH within their TH range.

To further explore the impact of treatment timing, we assessed whether there were differences in AH and HG between two subpopulations, consisting respectively of patients with early start of therapy (<7 years, also referred to as “subgroup A”) and late initiation (>7 years, “subgroup B”).

Children in subgroup A exhibited better H SDS during puberty and showed a trend toward higher AH SDS (*p* = 0.059), suggesting that earlier initiation of therapy may be associated with improved AH outcome, in line with previous reports [33,34]. Notably, baseline H SDS and Δ H-TH at early follow-up stages did not differ significantly between the two subgroups.

Consistently, both univariate and multivariate regression analyses identified age and H SDS at T0 as independent predictors of HG. These results emphasize the importance of early identification and timely referral of SGA children with persistent growth failure.

These findings have relevant clinical implications, highilighting the importance that prompt recognition may allow earlier initiation of rhGH therapy, thereby optimizing growth outcomes and improving the likelihood of achieving AH within the TH range.

Short SGA children who have not caught up in terms of growth by the age of 4 should be referred promptly for specialist assessment by general pediatricians, whose role in monitoring the growth progress of these patients is crucial [35].

However, the response to rhGH therapy remains highly variable. The term SGA encompasses a heterogeneous group of conditions, including diverse genetic backgrounds and alterations in the GH–IGF-I axis, many of which are not fully understood [36].

The indentification of these children born SGA who will benefit the most from rhGH treatment in terms of HG and AH remains a challenge [37].

Although several factors have been associated with better outcomes— absence of chromosomal abnormalities, maternal H within the normal range, greater first-year growth velocity, longer treatment duration, lower baseline IGF-I levels, later P0, and greater H at P0 [38]—no single predictor reliably identifies responders. Our findings partially confirm previous evidence, showing that H at P0 and maternal H are positively associated with AH, while advanced BA at T0 negatively impacts growth HG, likely reflecting reduced residual growth potential [37,38].

Furthermore, we asessed potential sex-related differences in growth patterns under rhGH treatment: in line with previous results, we found no significant gender-related differences in H SDS and BMI SDS at T0, T1, T2, P0, P1, P2, FH, nor in total H increase in SDS between males and females [39].

The impact of pubertal growth on AH in chidren born SGA has been widely investigated and analyzed in previously published studies; however, current evidence does not allow definitive conclusions regarding the overall pattern of pubertal development in this population [40].

It is now well established that children born SGA may experience an earlier onset of puberty than their AGA peers. It is important to emphasize that this earlier onset generally represents a relative advance rather than true precocious puberty, as puberty usually begins within the physiological age range [41], often due to rapid weight gain in early childhood, which leads to increased visceral adiposity, insulin resistance and elevated IGF-I concentrations [18,42]. Both the timing of puberty and the magnitude of the pubertal growth spurt are key determinants of AH outcomes [11].

Although pubertal development in SGA children is often clinically normal, several studies have reported a reduced pubertal HG and a shorter duration of peak H velocity compared with AGA peers. In addition, maximum growth velocity may be reached at an earlier pubertal stage, potentially resulting in earlier epiphyseal maturation and a reduced contribution of pubertal growth to AH [17,43].

In our cohort, pubertal development started within the normal physiological range and progressed regularly. As expected, girls entered puberty earlier than boys (*p* < 0.001). Under rhGH therapy, H deficit progressively decreased from T0 onward, with the greatest improvement occurring before P0.

However, despite ongoing rhGH therapy, patients entered puberty with a residual H deficit, with mean H SDS still close to −2.0 at P0. SGA patients in our cohort also did not exhibit a fully satisfactory pubertal growth spurt, and puberty did not result in further improvement in H SDS, with AH remaining below the TH range in most cases.

Notably, we observed that the greatest HG occurred during the prepubertal phase, whereas the contribution of puberty to THG was relatively modest. From P0 to AH, HG was limited, and a slight reduction in H SDS was observed. These findings suggest that, in SGA children, pubertal growth may not fully compensate for pre-existing H deficits [44].

These findings are clinically relevant, as H status at P0 is a key determinant of AH.

Patients in subgroup A achieved a better H SDS at P0 and seemed to reach higher AH compared with subgroup B, reinforcing the concept that optimizing prepubertal growth is essential [42,45].

These results are consistent with previous reports by Dahlgren et al., showing that initiating rhGH therapy more than two years before puberty is associated with improved AH outcomes, whereas delayed treatment reduces its effectiveness [31].

Importantly, in our cohort, rhGH treatment did not influence the timing of puberty, age at menarche, or progression of secondary sexual characteristics, all of which occurred within normal physiological limits.

The growth response following treatment with rhGH in short-statured SGA children differs markedly from that observed in children with GHD, who exhibit a constant growth rate, particularly during puberty. The pubertal growth spurt plays a key role in ensuring adequate H and contributes significantly to normal AH in patients with GHD, and usually does not differ from that observed physiologically in children [46]. Furthermore, children with GHD who start rhGH therapy early can derive the maximum benefit in terms of achieving near-normal AH. However, it is well known that if children with GHD enter puberty with short stature, they will be short as adults [47,48].

In our patients we also reported a progressive increase in HOMA-IR values during follow-up and specifically during puberty, with mean values approaching or slightly exceeding 2.5 at later stages.

Moreover, we did not observe clinically significant alterations in glucose metabolism or cases requiring treatment discontinuation of rhGH therapy, whose administration has been associated with a reduction in insulin sensitivity and a compensatory increase in acute insulin response (AIR) in previous published studies. Therefore, the observed changes are likely attributable to a combined effect of rhGH therapy and physiological pubertal insulin resistance and partially influenced by the well-known metabolic alterations observed in the SGA population [49,50].

We would like to highlight the usefulness and applicability of the findings of this study in pediatric clinical practice.

The main limitations of our study include the relatively small sample size and the retrospective design. Due to the retrospective design, it was not possible to conduct an a priori power calculation. However, a post hoc power analysis based on the observed difference in THG between groups and the pooled standard deviation yielded an estimated statistical power of approximately 70–75% at a two-sided α level of 0.05. While a significant difference was detected, the study may be underpowered to identify smaller yet clinically relevant effects.

Additional limitations include the potential for selection bias due to the retrospective design and the inclusion of patients who completed long-term follow-up to AH. Furthermore, the late referral for specialist assessment by primary healthcare services and the strict national regulatory criteria for rhGH treatment (AIFA Note 39) may have influenced the timing of therapy initiation, potentially limiting the generalizability of our findings to other healthcare settings. We acknowledge that data on cost-effectiveness and quality of life were not available in our cohort.

Future prospective studies are warranted to evaluate the broader clinical and socioeconomic impact of rhGH therapy in children born SGA and also to better clarify the pattern of pubertal growth and its contribution to AH in SGA patients treated with rhGH.

## 5. Conclusions

rhGH treatment in children born SGA with short stature is effective in the long term, allowing good statural recovery and a significant reduction in the gap between H at T0 and AH. Timing of rhGH initiation and primarily a prompt selection of SGA patients who do not catch up in growth and are elegible for rhGH treatment represents an important determinant in AH prognosis, but further studies are needed to confirm their influence on HG in SGA patients.

## Figures and Tables

**Table 1:** Characteristics of the study population at baseline.

	All
N. of patients (percentage)	49 (100)
n. of female (percentage)	25 (51)
n. of male (percentage)	24 (49)
Gestational age (weeks)	37.6 ± 3.0
Mean; range	38.0; 36.0–39.0
Birth weight (SDS)	−1.9 ± 0.9
Mean; range	−2.1; −2.5–1.7
Birth length (SDS)	−2.3 ± 0.8
Mean; range	−2.2; −2.8–−1.9
Target Height (SDS)	−1.23 ± 0.73
Mean; range	−1.21; −1.81–−1.02
Age at first visit (years)	5.6 ± 2.9
Mean; range	5.1; 3.2–7.9
Age at rhGH start (years)	7.3 ± 2.9
Mean; range	6.0; 3.6–8.75
Bone age at rhGH start	6.3 ± 2.9
Mean; range	6.8; 4.7–9.2
Height at rhGH start (SDS)	−3.08 ± 0.56
Mean; range	−2.98; −3.28–−2.69
Weight at rhGH start (SDS)	−3.25 ± 2.21
Mean; range	−2.95; −3.63–−2.27
BMI at rhGH start (SDS)	−1.20 ± 1.05
Mean; range	−1.09; −1.92–−0.54
rhGH dose at treatment start (µg /kg/day)	0.035 ± 0.01
Mean; range	0.035; 0.034–0.036

**Table 2:** Evolution of height, weight, BMI, and Δ H-TH during follow-up of SGA patients treated with rhGH expressed as mean ± SDS.

Parameter	T0	T1	T2	P0	P1	P2	AH
Height (SDS)	−3.08 ± 0.56	−2.53 ± 0.55	−2.18 ± 0.54	−1.99 ± 0.64	−1.67 ± 0.76	−1.39 ± 0.93	−1.75 ± 0.63
Weight (SDS)	−3.25 ± 2.2	−2.50 ± 0.94	−2.02 ± 0.98	−1.68 ± 0.95	−1.34 ± 1.07	−1.13 ± 1.05	−1.10 ± 1.14
BMI (SDS)	−1.2 ± 1.05	−2.17 ± 6.47	−1.21 ± 1.13	−0.89 ± 1.04	−0.80 ± 0.94	−0.81 ± 0.98	−0.56 ± 1.28
DELTA H-TH (SDS)	−1.81 ± 0.80	−1.26 ± 0.81	−0.96 ± 0.82	−0.75 ± 0.90	−0.43 ± 1.04	−0.14 ± 1.18	−0.45 ± 0.79

Abbreviations: T0: start of rhGH therapy; T1: one year after the start of rhGH therapy; T2: two years after the start of rhGH therapy; P0: onset of puberty; P1: one year after the onset of puberty; P2: two years after the onset of puberty; AH: adult height; BMI: body mass index; Δ H-TH: difference between the child’s height and the family target height; SDS: standard deviation score.

**Table 3:** IGF-1 levels and glycemic metabolism indicators (insulin, fasting glucose and HOMA score) during follow-up of SGA patients treated with rhGH expressed as mean ± SD.

	T0	T1	T2	P0	P1	P2
IGF-1 (ng/mL)	125 ± 63	233 ± 98	299 ± 133	325 ± 140	391 ± 138	445 ± 168
Insulin (mcrUI/mL)	5.1 ± 3.2	7.8 ± 5.3	9.5 ± 6.3	9.8 ± 5.8	11.2 ± 4.7	11.6 ± 5.5
Fasting Glucose (mg/dL)	83 ± 13	83 ± 11	86 ± 9	89 ± 6	90 ± 7	88 ± 6
HOMA score	1.07 ± 0.71	1.72 ± 1.31	2.14 ± 1.46	2.19 ± 1.39	2.61 ± 1.01	2.53 ± 1.23

Abbreviations: T0: start of rhGH therapy; T1: one year after the start of rhGH therapy; T2: two years after the start of rhGH therapy; P0: onset of puberty; P1: one year after the onset of puberty; P2: two years after the onset of puberty; IGF-1: insulin growth factor 1.

**Table 4:** Spearman correlation analyses.

	HG (SDS)	AH (SDS)	Height (SDS) at P0
**Target height** (SDS)	0.333	0.433	0.2018
*(rho; p value)*	*n.s.*	0.0099	*n.s.*
**Maternal height** (SDS)	0.3278	0.4964	0.1568
*(rho; p value)*	*n.s.*	0.0027	*n.s.*
**Father height** (SD)	−0.0626	0.1591	0.1982
*(rho; p value)*	*n.s.*	*n.s.*	*n.s.*
**Age at T0** (years)	0.2974	−0.1249	−0.7320
*(rho; p value)*	*n.s.*	*n.s.*	0
**Bone age at T0 (years)**	−0.3548	−0.1897	−0.7004
**(rho; *p* value)**	0.0342	n.s.	0
**Age at P0 (years)**	−0.2073	−0.1687	−0.4642
**(rho; *p* value)**	n.s.	n.s.	0.0036
**Height (SD) at P0**	0.2953	0.4933	1
**(rho; *p* value)**	n.s.	0.0019	----
**Duration of rhGH treatment** (years)			
*(rho; p value)*	0.2634	0.1986	0.5788
	*n.s.*	*n.s.*	0.0002

Abbreviations: HG: height gain; AH: adult height; T0: start of rhGH therapy; P0: onset of puberty, n.s.: not significant.

**Table 5:** Univariate and multivariate regression analysis for HG.

Variable Influencing HG (SDS)		Univariate Regression	Multivariate Regression
**Age at rhGH start**	Β	−0.08	−0.100
	95% IC	−0.14 to −0.016	−0.186 to −0.013
	*p value*	0.014 *	0.026 *
**Height SDS at T0**	Β	−0.584	−0.853
	95% IC	−0.898 to −0.269	CI −1.625 to −0.080
	*p value*	0.001 **	0.032 *

Abbreviations: rhGH: recombinant human growth hormone; T0: start of rhGH treatment, HG: height gain. * *p* < 0.05, ** *p* < 0.005.

## Data Availability

The data presented in this study are available on request from the corresponding author due to privacy and ethical restrictions.

## Abbreviations

The following abbreviations are used in this manuscript:

SGA	Small for Gestational Age
H	Height
HG	Height gain
GT	Growth trajectory
AH	Adult height
rhGH	Recombinant human growth hormone
Delta H-TH	Delta within height and target height
GHD	Growth hormone deficiency
TH	Target height
BMI	Body mass index
BA	Bone age
THG	Total height gain
AGA	Adequate for gestational age
